# Harnessing nucleotide metabolism and immunity in cancer: a tumour microenvironment perspective

**DOI:** 10.1111/febs.17278

**Published:** 2024-09-22

**Authors:** Hadil Suleiman, Alexandra Emerson, Peter M. Wilson, Karl A. Mulligan, Robert D. Ladner, Melissa J. LaBonte

**Affiliations:** ^1^ Patrick G Johnston Centre for Cancer Research Queen's University Belfast UK; ^2^ CV6 Therapeutics (NI) Ltd Belfast UK

**Keywords:** cancer immunology, immune checkpoint inhibitors, immunometabolism, metabolic therapies, nucleotide metabolism, purine metabolism, pyrimidine synthesis, therapeutic modulation, tumour microenvironment

## Abstract

The tumour microenvironment (TME) is a dynamic nexus where cancer cell metabolism and the immune system intricately converge, with nucleotide metabolism (NM) playing a pivotal role. This review explores the critical function of NM in cancer cell proliferation and its profound influence on the TME and immune landscape. NM is essential for DNA and RNA synthesis and is markedly upregulated in cancer cells to meet the demands of rapid growth. This metabolic rewiring fuels cancer progression, but also shapes the TME, impacting the function and viability of immune cells. The altered nucleotide milieu in the TME can suppress immune response, aiding cancer cell evasion from immune surveillance. Drug discoveries in the field of NM have revealed different therapeutic strategies, including inhibitors of nucleotide synthesis and drugs targeting salvage pathways, which are discussed thoroughly in this review. Furthermore, the emerging strategy of combining NM‐targeted therapies with immunotherapies is emphasised, particularly their effect on sensitising tumours to immune checkpoint inhibitors and enhancing overall treatment efficacy. The Human Genome Project paved the way for personalised medicine, countering the established ‘one size fits all’ approach to cancer treatment. Advances in understanding the TME and NM have spurred interest in personalised therapeutic strategies. This review highlights the potential of leveraging individual tumour metabolic profiles to guide treatment selection, aiming to optimise efficacy and minimise adverse effects. The strategic importance of targeting NM in cancer therapy and its synergistic potential with immunotherapies offers a path towards more effective and personalised cancer treatments.

## Graphical abstract

This review explores how changes in nucleotide metabolism affect the tumour microenvironment and immune responses in cancer. Here, we discuss the dual role of nucleotide metabolism in promoting cancer cell growth and shaping immune suppression. We highlight how therapies targeting nucleotide pathways can enhance immunotherapy efficacy, offering new strategies for more effective and personalised cancer treatments. Figure created using Biorender.com.

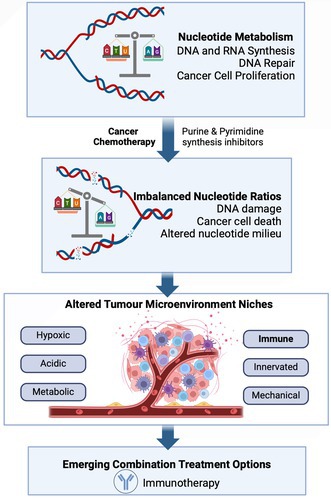

**Abbreviations**
5‐FU: 5‐fluorouracil
5‐PRA: 5‐phosphoribosylamine
A2A: adenosine A2A receptor
ADP: adenosine diphosphate
ADSS: adenylsuccinate synthetase
ALL: acute lymphoblastic leukaemia
AMP: adenosine monophosphate
APCs: antigen‐presenting cells
Ara‐C: cytarabine
Asp: aspartate
ATCase: aspartate transcarabmylase
ATP: triphosphate
CAD: phosphate synthetase 2, aspartate transcarbamylase, and dihydroorotase
CD: cluster of differentiation
cGAS: cyclic GMP‐AMP synthase
CMP: cytosine monophosphate
CMPK: cytidine monophosphate kinase
CRC: colorectal cancer
CTL: cytotoxic T‐lymphocyte
CTP: cytidine triphosphate
CTPS: CTP synthase
d: ‘deoxy’
DC: dendritic cell
DHFR: dihydrofolate reductase
DHO: dihydroorotase
DHODH: dihydroorotate dehydrogenase
DNA: deoxyribonucleic acid
dNTP: deoxynucleoside triphosphate
DPD: dihydropyrimidine dehydrogenase
dUTPase: deoxyuridine triphosphate nucleotidohydrolase
ECM: extracellular matrix
ESCC: oesophageal squamous cell carcinoma
FOXP3: forkhead box P3
FUdR: floxuridine
GART: glycinamide ribonucleotide transformylase
GDP: guanine diphosphate
GEC: gastroesophageal cancer
Gln: glutamine
GMP: guanine monophosphate
HGPRT: hypoxanthine‐guanine phosphoribosyltransferase
HIF‐1: hypoxia‐inducible factor‐1
ICB: immune checkpoint blockade
ICD: immunogenic cell death
ICG: immune checkpoint gene
ICI: immune checkpoint inhibitor
IDO: indoleamine 2,3‐dioxygenase
IFN: interferon
IL: interleukin
IME: immune microenvironment
IMP: inosine monophosphate
IMPDH: inosine monophosphate dehydrogenase
IPS: immunophenoscore
LDH: lactate dehydrogenase
LLC: Lewis lung carcinoma
MCT: monocarboxylate transporters
MDSC: myeloid‐derived suppressor cell
Mena: mammalian enabled
MHC: major histocompatibility complex
MMP: matrix metallopeptidase
MSI: microsatellite instable
MSS: microsatellite stable
mtDNA: mitochondrial deoxyribonucleic acid
NDPK: nucleotide diphosphate kinase
NF‐κB: nuclear factor kappa‐light‐chain‐enhancer of activated B cells
NK: natural killer
NM: nucleotide metabolism
NSCLC: non‐small cell lung cancer
NTP: nucleoside triphosphate
PALA: *N*‐phosphonoacetyl‐l‐aspartate
PD‐1: programmed death protein‐1
PD‐L1: programmed death ligand‐1
PNP: purine nucleoside phosphorylase
PRPP: phosphoribosyl pyrophosphate
PTMB: pyrimidine‐rich transversion mutational bias
RNA: ribonucleic acid
RNR: ribonucleotide reductase
ROS: reactive oxygen species
SLC25A1: solute carrier family 25 member 1
STING: stimulator of interferon genes
TAM: tumour‐associated macrophage
T‐bet: T‐box expressed in T‐cells
TBK1: TANK‐binding kinase 1
TDP: thymidine diphosphate
TGF‐β: transforming growth factor‐β
Th‐1: T‐helper cell 1
TIL: tumour‐infiltrating lymphocyte
TIP: tumour immunophenotype
TK1: thymidine kinase 1
TLR: toll‐like receptor
TMB: tumour mutation burden
TME: tumour microenvironment
TMP: thymidine monophosphate
TMPK: TMP kinase
TNF‐α: tumour necrosis factor‐α
Tregs: T‐regulatory cells
TS: thymidylate synthase
TTP: thymidine triphosphate
UCD: urea cycle dysregulation
UCK1: uridine‐cytidine kinase 1
UDP: uridine diphosphate
UMP: uridine monophosphate
UMPS: UMP synthetase
UTP: uridine triphosphate
VEGF: vascular endothelial growth factor
WGCNA: weighted gene co‐expression network analysis

## The tumour microenvironment

**Fig. 1:**
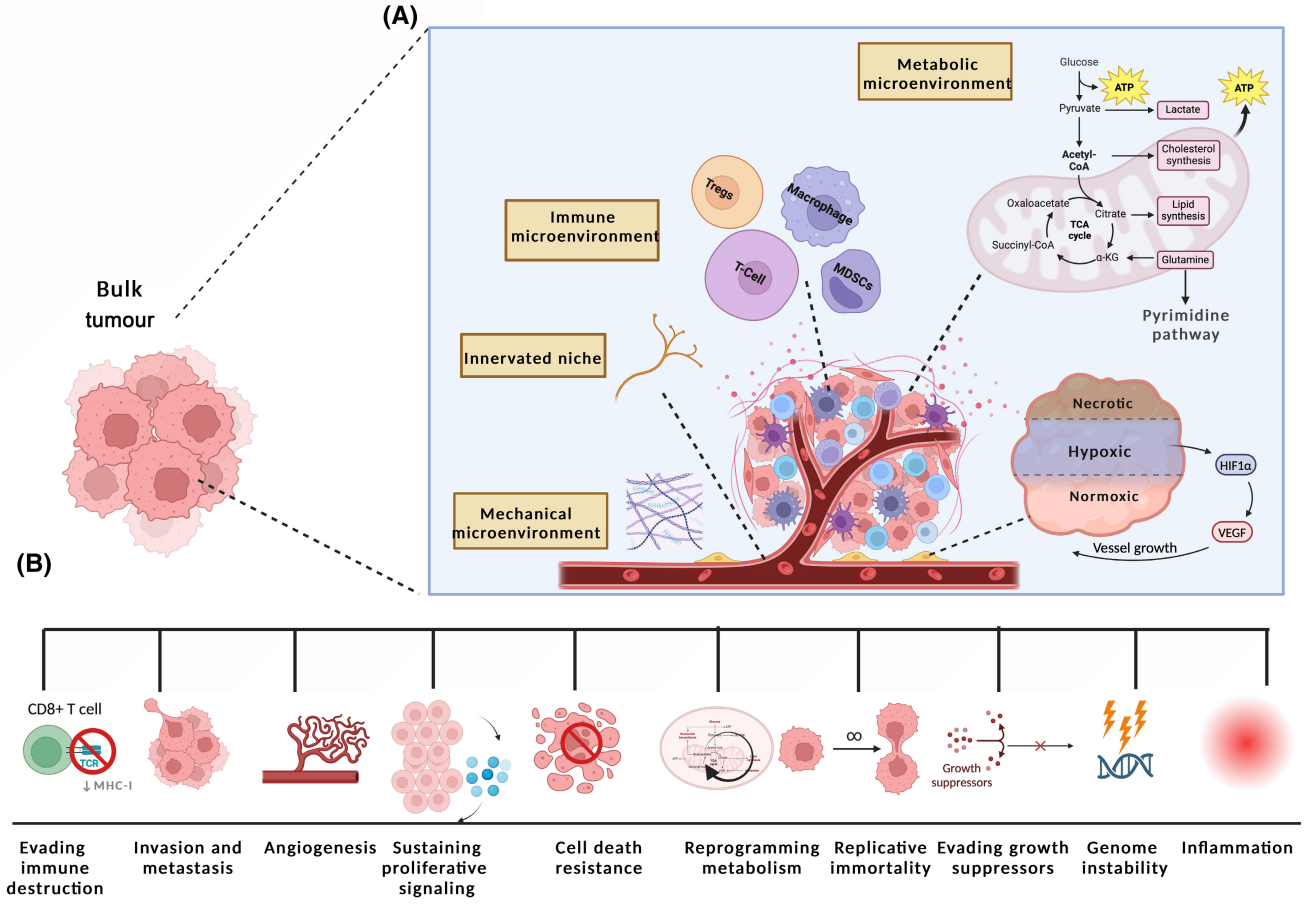
The hallmarks and enabling characteristics of cancer within the tumour microenvironment (TME). An illustration of the hallmarks of cancer and enabling characteristics of TME, identified by Hanahan and colleagues in 2000–2011 [2, 3, 4], created with BioRender.com. (A) The TME is depicted as an intricate network composed of specialised metabolic, immune, innervated, mechanical, and hypoxic microenvironments, each contributing to cancer progression through different mechanisms. At the centre, we see the depiction of the metabolic microenvironment, which includes the Warburg effect, indicating a preference for glycolysis over oxidative phosphorylation even in the presence of oxygen. This metabolic shift results in lactate production and is linked to various aspects of cancer progression, such as angiogenesis, promoted by hypoxia‐inducible factor 1 (HIF1) and vascular endothelial growth factor (VEGF). Surrounding the central illustration are different niches, including the IME populated by T‐cells, regulatory T‐cells (Tregs), macrophages, and myeloid‐derived suppressor cells (MDSCs), each playing distinct roles in modulating immune responses within the TME. The innervated niche refers to the network of nerves that can influence tumour growth, while the mechanical microenvironment includes the extracellular matrix (ECM) and its impact on cell behaviour and cancer progression. The pyrimidine pathway is highlighted, emphasising its role in cancer cell proliferation and survival. The TME's complexity is further illustrated by the hypoxic and necrotic areas, which are consequences of rapid cancer cell growth outpacing blood vessel development. (B) Below the central illustration is a depiction of the cancer progression stages, highlighted by various cellular and molecular processes such as evading immune destruction, invasion and metastasis, angiogenesis, and resistance to cell death. ATP, adenosine triphosphate; ECM, extracellular matrix; HIF1, hypoxia‐inducible factor 1; MDSCs, myeloid‐derived suppressor cells; MHC‐I, major histocompatibility complex class I; TCA, tricarboxylic acid cycle; Tregs, regulatory T‐cells; VEGF, vascular endothelial growth factor; α‐KG, alpha‐ketoglutarate. Figure adapted from Refs [2, 3, 4].

For decades cancer research has focused on the seeds rather than the soil, an idea that was proposed by Stephen Paget in 1889, who hypothesised that metastasis in cancer would not occur at random but would only arise in a fertile environment [1]. The soil analogy depicts what is currently known as the tumour microenvironment (TME). Furthermore, the significant work of Hanahan and colleagues two decades ago in characterising the hallmarks of cancer is widely recognised in the world of cancer research and served as a foundation for many researchers in the field [2]. Hanahan published an updated landscape of the hallmarks of cancer in 2011 [2], in which the role of the TME in supporting immune evasion was highlighted, with tumour‐promoting inflammation and genome instability identified as ‘enabling characteristics’ [2, 3, 4] (Fig. 1).

The TME is a niche of heterogenous cell populations including tumour‐infiltrating immune cells, blood vessels, stromal cells and the ECM (Fig. 1). The TME's composition varies depending on the tumour type in which it is created, and is recruited to induce a network of molecular and physical alterations in their host tissue to encourage tumour growth [5]. In addition, the TME and its immunosuppressive elements play a crucial role not only in tumour progression, but to the response to different cancer treatments, therefore many researchers are focusing on modifying the TME to enhance the efficacy of cancer therapies [6]. This peculiar link between the host and cancer cells is primarily composed of six specialised microenvironments that collaborate to support tumour growth and represent an attractive target for anti‐tumour combination therapy. They comprise of hypoxic niches, acidic niches, innervated and mechanical niches, metabolic niches and immunological niches [7, 8].

### Hypoxic niche

Hypoxia in the TME is largely mediated by the cellular response to oxygen deficiency, which is orchestrated by HIFs [9]. Acute and chronic hypoxia influence tumour behaviour and the cancer cell phenotype in distinct ways, with acute hypoxia being associated with increased survival, autophagy, and the selection of cells with cancer stem cell characteristics, while chronic hypoxia may contribute to tumour regression in certain contexts [10, 11].

Hypoxia prompts genomic instability, heightening mutation rates and DNA damage, thereby fostering oncogenic activation and metastatic evolution. It also supports cancer stem cell maintenance, linked to tumour aggressiveness and treatment evasion. Dysfunctional angiogenesis under hypoxia leads to poorly structured vessels, hindering effective drug delivery and furthering heterogeneity within the TME [10]. Combating hypoxia remains key, with strategies targeting the hypoxic microenvironment and its associated cancer stem cells, alongside chemotherapy, promising a more efficacious approach to cancer treatment [12].

### Acidic niche

The acidic microenvironment in cancer and the TME is characterised by an extracellular pH that is lower than normal body tissues, a condition resulting from the cancer cells' altered metabolism [7]. This microenvironmental acidity is not solely due to hypoxia; even cells at the tumour surface can undergo metabolic shifts towards aerobic glycolysis, producing lactic acid and contributing to the acidic conditions [13]. This shift in metabolism is widely known as the Warburg effect [14], which not only favours cancer cell survival and proliferation, but also promotes invasion and metastasis by influencing oncogenic, pro‐survival gene expression related to these processes [15].

The acidic conditions within the TME are known to affect various cellular functions, modulating the activity of enzymes and altering the expression and splicing of genes involved in cell migration, such as Mammalian enabled (Mena) and CD44 [16]. These genes help cancer cells migrate and invade surrounding tissues, facilitating tumour spread. Furthermore, the acidity of the TME can suppress immune responses, which further contributes to tumour progression and may impact the effectiveness of immunotherapies [17]. Interestingly, interventions that modulate the pH of the TME, such as the administration of sodium bicarbonate in animal models [18, 19, 20], have been shown to reduce tumour acidity, shift gene expression towards a more normal state, and decrease metastasis. While sodium bicarbonate itself may not be a feasible treatment for humans due to tolerance issues, targeting TME acidity could be a valuable approach to cancer therapy.

### Innervated and mechanical niches

Innervated and mechanical niches affect tumour progression and metastasis in a structural landscape. During angiogenesis and vascular formation, the tumour cells can induce the formation of nerve fibres (axonogenesis), where the increased nerve density promotes tumour growth [21]. In contrast, several studies showed that denervation reduces tumour growth [22]. Research indicates that disrupting cancer cell‐derived exosomes, which promote axonogenesis, might be a viable therapeutic approach [22, 23, 24]. The mechanical niche within the TME plays a pivotal role in cancer progression and metastasis, underscored by changes in ECM stiffness, intercellular fluid pressure, and solid stress [25]. Mechanical imbalances promote metastasis and tumour progression for instance, where ECM stiffening, through increased collagen deposition and cross‐linking, facilitates tumour aggression and affects immune cell behaviour, resulting in tumour aggression and increased immune cell infiltration [7, 26]. Mechanotransduction pathways activated by ECM changes, such as integrin signalling and the YAP/TAZ axis, promote cancer cell metastasis and stemness [27, 28]. The interaction between cancer cells and cancer‐associated fibroblasts (CAFs) exacerbates ECM remodelling, enhancing tumour growth [29]. Targeting ECM stiffness and mechanotransduction presents a novel therapeutic approach, suggesting the biomechanical properties of the TME as crucial for cancer dynamics and treatment outcomes.

### Metabolic niche

The metabolic microenvironment within the TME is intricately linked to cancer sustainability and progression of cancer cells. The Warburg effect, a hallmark of cancer metabolism, illustrates how cancer cells preferentially metabolise glucose to lactate even in the presence of oxygen. This metabolic reprogramming supports rapid cancer cell growth by enhancing glycolysis over oxidative phosphorylation, leading to the accumulation of biosynthetic precursors for macromolecules like nucleotides, amino acids, and lipids [8, 12]. The role of lactate extends beyond a mere metabolic by‐product; it is pivotal in angiogenesis, the adaptation to hypoxic conditions, and the establishment of an immunosuppressive TME, primarily by modulating the behaviour of immune cells towards a pro‐tumour phenotype [7]. Metabolites, including reactive oxygen species (ROS), play dual roles in redox balance and cell signalling, affecting epigenetics and proliferation [7, 26]. Furthermore, the noncanonical roles of enzymes like PGK1, including regulation of cell growth, survival, proliferation and mediating treatment response, underscore metabolism's significance in cancer [30]. Cancer cells communicate with and reshape their microenvironment via exosomes carrying metabolic and genetic information, reprogramming neighbouring cells and immune response [31]. Understanding these interactions is crucial for developing more effective cancer treatments [32].

### Immunological niche

The immune microenvironment (IME) plays a pivotal role in cancer maintenance and progression. It encompasses a dynamic interplay between various immune cells, including adaptive and innate immune cells, which can both suppress and promote tumour growth. However, cancer cells commonly develop adaptive techniques to hijack and avoid immune system surveillance, creating opportunities to intervene with immunotherapy [33]. Adaptive immunity, characterised by T‐cells, natural killer (NK) cells and B‐cells, is an immune response that evolves in response to specific antigens and is crucial for long‐term immune memory and the precise targeting of tumour cells [34]. Innate immunity, however, remains largely non‐specific and involves macrophages, dendritic cells (DCs) and neutrophils which provide an immediate response to threats and are essential for initiating and modulating adaptive immune responses [5, 33, 35]. Cancer cells often exploit the underlying biology of these immune mechanisms to avoid immune detection and destruction. One key example of how they achieve this is by promoting the differentiation of immune‐suppressive cell types like Tregs and Myeloid‐derived suppressor cells (MDSCs), which effectively dampen the immune anti‐tumour response [6]. Table 1 summarises the roles of key immune cells in the TME, highlighting their role as tumour‐promoting or tumour‐suppressing, or both, depending on the stage of tumour development.

**Table 1:** Classification of immune cells inhabiting the tumour microenvironment [8, 33]. CTLs, cytotoxic T‐cells lymphocytes; DCs, dendritic cells; ECM, extra cellular matrix; IFN‐γ, interferon‐γ; IL‐2, interleukin‐2; NK, natural killer cells; ROS, reactive oxygen species; TAMS, tumour associated macrophages, cells; Th‐1, T‐helper cell 1.

Immune cell type	Sub‐types	Function	Cancer cell growth
T‐cells	CD8^+^	CD8^+^ detect tumour antigens and target them for destruction and secretion of IFN‐γ to suppress angiogenesis	Suppress
	CD4^+^	CD4^+^, differentiate into T‐helper 1 (Th‐1) and secret IL‐2 and IFN‐γ to activate CD8^+^	
NK		Scan the blood stream for viruses and tumour cells Destroy cancer cells directly by secreting perforin and granzymes or by producing inflammatory cytokines	Suppress
Neutrophils		Release cytokines and ROS to promote inflammation and cancer apoptosis Induce angiogenesis in later stages of cancer development	Suppress/Promote
Macrophages (TAMs)	M1‐polarised	M1‐polarised has an anti‐tumour activity which kill cells by producing cytokines and ROS	Suppress
	M2‐polarised	M2‐polarised promote tumour growth by promoting angiogenesis and ECM remodelling besides its function in suppressing immunosurveillance and cytokines activity	Promote
DCs		Antigen presentation and induce immunity activation by increasing tumour infiltration	Suppress/Promote
B cells		Antigen presentation, promote cytokines secretion and anti‐tumour antibody production Induce a pro‐tumour function through promoting tumour progression via the secretion of immune suppressive cytokines, suppressing cytotoxic T‐lymphocytes (CTLs) function and inducing angiogenesis	Suppress/Promote

T‐cells are one of the key immune cell types in the TME. There are a number of sub‐populations of T‐cells based on their activation status and function and includes CD8^+^ T‐cells, CD4^+^ T‐cells and Tregs [6]. CD8^+^ T‐cells exhibit cytotoxic activity through recognising aberrant antigens on the surface of cancer cells and secreting enzymes to destroy the cells. CD8^+^ T‐cells additionally possess anti‐angiogenic effects by secreting interferon‐gamma (IFN‐γ) that inhibits endothelial cell growth and blocks capillary formation [36].

### Immune evasion and suppression mechanisms

Tregs and MDSCs are major players in cancer development [6]. Tregs are a subpopulation of CD4^+^ cells that express forkhead box P3 (FOXP3) and are responsible for immune homeostasis and suppress overactive immunological responses [8]. Tregs play critical role in assisting CD8^+^ activation and proliferation, as well as limiting autoimmunity and dampening inflammatory responses. However, within the TME, they promote cancer progression by inhibiting anti‐tumour responses through IL‐2 secretion which modulates NK cells, impairing function, or directly by inhibiting antigen presenting cells (APCs) resulting in blockade of cytokines secretion and hence T cell activation [37]. Treg infiltration is increased in malignancies, accounting for 10–50% of CD4^+^ cells. Furthermore, high levels of Tregs compared to other T‐cell types predict a poor prognosis in a variety of malignancies including ovarian cancer [8], whereas elevated levels of FOXP3 (non‐Treg cells), in parallel with high amounts of inflammatory cytokines in malignancies like colorectal cancer (CRC) indicate a positive prognosis [38].

Myeloid‐derived suppressor cells are a heterogeneous population of myeloid cells that exhibit substantial immunosuppressive activity in both innate and adaptive immunity. Similar to Tregs, high levels of MDSC infiltration in tumours is associated with a poor prognosis. A study on metastatic CRC found that the accumulation of MDSCs resulted in a poor outcome of the combination regimen of 5‐FU, oxaliplatin, and bevacizumab [39, 40]. MDSCs protect cancer cells by suppressing immunosurveillance, promoting angiogenesis via the secretion of matrix metallopeptidase 9 (MMP9) and the vascular endothelial growth factor (VEGF), and inducing metastasis [8, 41].

### Tumour immune profiles: from ‘cold’ to ‘hot’

According to the IME profile, tumours are classified as either immune‐inflamed (‘hot’) or immune‐excluded and immune desert (‘cold’), based on their immune characteristics. Cold tumours are marked by diminished T‐cell infiltration, low tumour mutational burden (TMB), reduced MHC‐I expression [41]. These factors contribute to the immunologically ‘cold’ phenotype, which is often accompanied by low PD‐L1 levels [41]. Immune‐excluded tumours contain CD8^+^ T‐cell clusters that fail to infiltrate the tumour effectively, in contrast to immune desert tumours, which lacks CD8^+^ T‐cells entirely. Moreover, ‘cold’ tumours harbour immunosuppressive cells, including TAMs, Tregs, and MDSCs, impeding anti‐tumour responses [41, 42].

### Strategies to modulate the IME for enhanced immunotherapy

Conversely, ‘hot’ tumours exhibit elevated T‐cell infiltration, robust IFN‐γ signalling, and higher TMB, making them more amenable to immunotherapy [42]. These active immune processes within the TME often lead to elevated PD‐L1 expression as a feedback mechanism [43, 44]. Strategies to convert ‘cold’ tumours to ‘hot’ tumours – thereby enhancing their response to immune checkpoint blockade (ICB) therapy and improve patient outcomes – focus on increasing T‐cell infiltration and activation [45]. Methods include promoting immunogenic cell death (ICD) for T‐cell priming [46] via oncolytic viruses [47], radiotherapy [48], chemotherapy [49], and toll‐like receptor (TLR) agonists treatments [49], alongside T‐cell expansion through cancer vaccines [50]. Furthermore, anti‐angiogenic therapies, transforming growth factor‐beta (TGFβ) inhibitors [51], CXCR4 inhibitors and epigenetic modification inhibitors [52] are employed to improve T‐cell trafficking and infiltration into tumours. The IME's complexity is further underscored by its ability to form ectopic lymphoid structures, akin to secondary lymphoid organs, within tumours. These structures can facilitate local immune responses against the tumour, yet their full potential in cancer therapy remains to be explored [53]. Moreover, the hypoxia prevalent within tumours can lead to a metabolic reprogramming of both cancer and immune cells, impacting immune surveillance and therapy response.

### Crosstalk and metabolic influences in the IME

Recent research emphasises the importance of understanding the crosstalk between different components of the IME, including the effects of metabolic changes and hypoxia, and how these factors can influence the effectiveness of immunotherapies like checkpoint inhibitors. There is a growing interest in exploring novel methods to harness the IME to enhance anti‐tumour immunity, such as targeting specific immune cell populations, modulating the metabolic landscape, or disrupting immunosuppressive networks within the tumour.

As has been shown, the TME plays a pivotal role in cancer progression and treatment response, largely through its complex interplay of hypoxic, acidic, innervated, mechanical, metabolic, and immunological niches. Each of these specialised microenvironments contributes to the unique cellular and molecular landscape that supports tumour growth and mediates resistance to therapies. NM alterations in cancer are closely linked to these microenvironmental factors, impacting both tumour cell proliferation and the immune landscape.

## Nucleotide metabolism

Maintaining intracellular nucleotide pools is essential for cell growth, metabolism and proliferation. In normal dividing cells, NM is tightly regulated to maintain correct balances of pyrimidine nucleotides (cytosine, thymine and uracil) and purine nucleotides (adenine and guanine) [54]. These five nucleotides are basic units of deoxyribonucleic acid (DNA) and ribonucleic acid (RNA), which are required for cellular function and proliferation. Cancer cells often manipulate NM pathways to support enhanced proliferation rates and tumour progression, therefore understanding the intricacies of NM under health and diseased states has been paramount for understanding cancer progression, therapeutic resistance and developing effective treatment strategies [55].

Nucleotide pools are expanded and maintained in a homeostatic manner by two biosynthetic pathways [56]: (a) *de novo* synthesis from sugar or amino acid precursors and (b) nucleotide salvage from nucleic acid turnover or dietary consumption [57].

*De novo* purine synthesis was first described by Buchannan and Hartman in 1959 [58] and begins with ribose‐5‐phosphate, a pentose sugar produced from the breakdown of glucose in the pentose phosphate metabolic pathway [59]. Ribose‐5‐phosphate is converted to phosphoribosyl pyrophosphate (PRPP) and a glutamine‐derived amine group is then added to form 5‐phosphoribosylamine (5‐PRA). Subsequent ATP‐dependent reactions then convert 5‐PRA to purine precursor inosine monophosphate (IMP), involving several key catalysts such as trifunctional enzyme glycinamide ribonucleotide transformylase (GART) [60]. IMP dehydrogenase (IMPDH) and adenylosuccinate synthetase (ADSS) serve as key enzymes in the conversion of IMP to guanine and adenine monophosphate (GMP and AMP) respectively [61]. The *de novo* purine synthesis pathway was recently reviewed in detail [62].

**Fig. 2:**
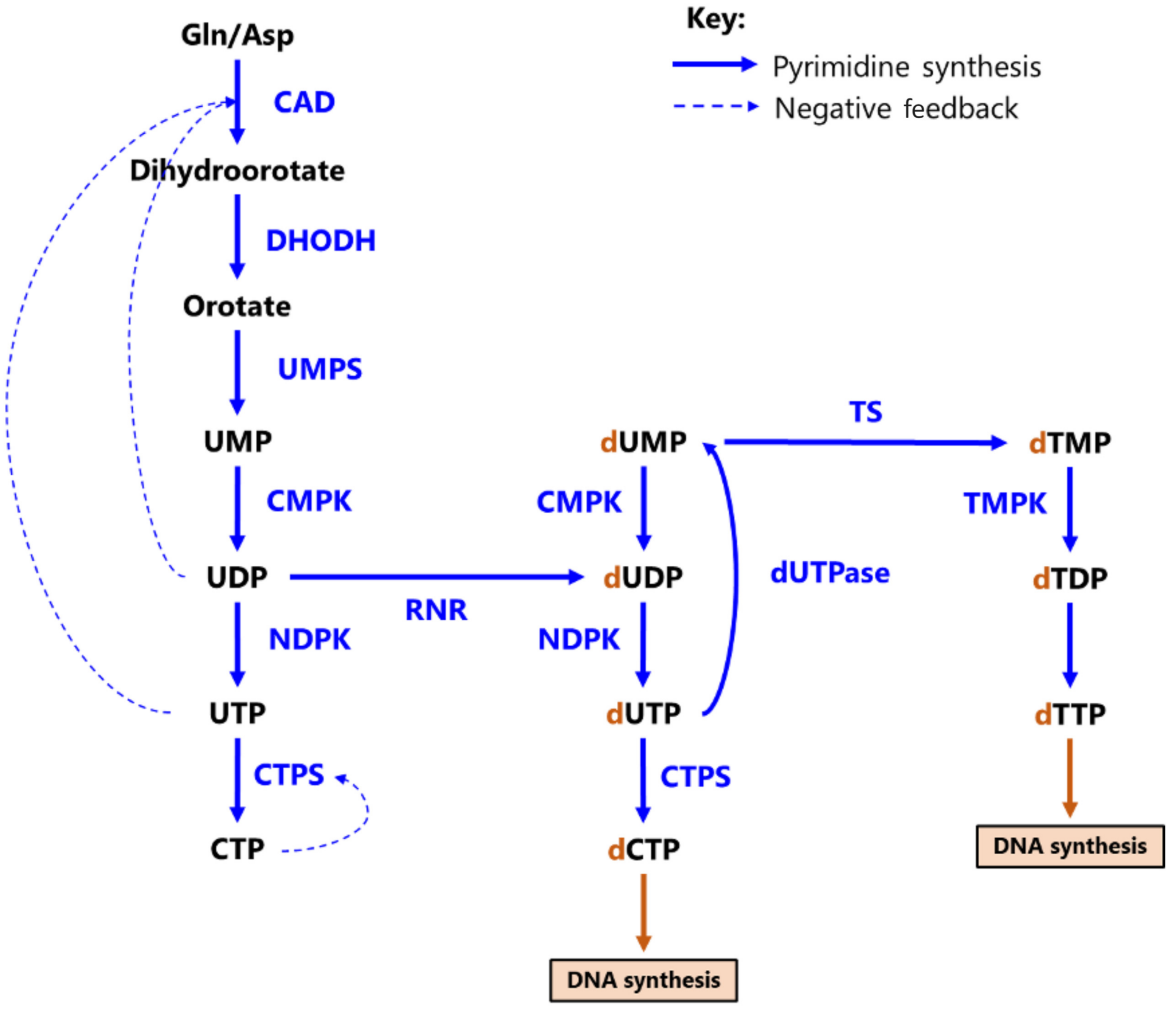
*De novo* pyrimidine synthesis. Summary pathway depicting key reactions within *de novo* pyrimidine synthesis. Intermediates are shown in black, whilst metabolic enzymes are shown in blue, with negative feedback loops shown by dotted blue lines. Ribonucleotide reductases (RNR) catalyse the conversion of nucleoside diphosphates (NDPs) to deoxynucleotide diphosphates (dNDPs; indicated by orange ‘d’). Cytidine triphosphate (CTP) negatively feeds back on CTP Synthetase (CTPS), whilst uridine diphosphate (UDP) and uridine triphosphate (UTP) negatively feedback on carbamoyl‐phosphate synthetase 2 aspartate transcarbamylase and dihydroorotase (CAD). Asp, aspartate; CAD, carbamoyl‐phosphate synthetase 2 aspartate transcarbamylase and dihydroorotase; CMPK, cytidine monophosphate kinase; CTP, cytidine triphosphate; CTPS, cytidine triphosphate synthase; d, ‘deoxy’; DHODH, dihydroorotate dehydrogenase; DUT, deoxyuridine 5′‐triphosphate nucleotidohydrolase; dUTPase, deoxyuridine triphosphate nucleotidohydrolase; Gln, glutamine; NDPK, nucleotide diphosphate kinase; TDP, thymidine diphosphate; TMP, thymidine monophosphate; TMPK, thymidine monophosphate kinase; TS, thymidylate synthase; TTP, thymidine triphosphate; UDP, uridine diphosphate; UMP, uridine monophosphate; UMPS, uridine monophosphate synthetase; UTP, uridine triphosphate

*De novo* pyrimidines are assembled through a 6‐step pathway beginning with the l‐glutamine and l‐aspartate amino acids (Fig. 2). The first three reactions, including the rate‐limiting step, are catalysed by carbamoyl‐phosphate synthetase 2 (CAD), aspartate transcarbamylase (ATCase) and dihydroorotase (DHO) [63, 64]. The product of these reactions is dihydroorotate, which is catalysed to orotate by dihydroorotate dehydrogenase (DHODH) via the mitochondrial electron transport chain [65]. Uridine monophosphate synthetase (UMPS) then converts orotate to uridine‐5‐phosphate (UMP) in a two‐step process, which can be phosphorylated to UDP, the precursor of deoxycytidine and deoxythymidine pyrimidines [66].

Purine and pyrimidine salvage pathways utilise free nucleobases and nucleosides recycled from degraded nucleic acids or nucleotides absorbed from the bloodstream via nucleoside transporter proteins [67]. Purine salvage is catalysed by hypoxanthine‐guanine phosphoribosyltransferase (HGPRT) and adenine phosphoribosyltransferase to generate GMP and AMP respectively, whilst pyrimidine synthesis is catalysed by uridine‐cytidine kinases 1 and 2 (UCK1/2) to generate UMP and CMP. TMP may be synthesised from UMP, or from salvaged thymidine via thymidine kinases 1 and 2 in ATP‐dependent reactions [64].

Nucleotide degradation is another crucial element in the homeostasis of nucleotide pools, since relative abundance of nucleotides is key for DNA replication, cell cycle control and cell division [68]. Nucleotide pools are regulated by negative feedback loops at both the enzyme and substrate levels. Purine metabolites GMP and AMP signal negative feedback through interaction with PRPP synthetase [69], and are degraded via conversion to their relative nucleoside via nucleotidase enzymes. Pyrimidine negative feedback occurs through CAD binding, mediated by UTP signalling, with CTP synthase regulating both UTP and CTP pools [70]. Pyrimidine degradation occurs through dephosphorylation to the relative monophosphate form and is largely regulated by dihydropyrimidine dehydrogenase (DPD) [71].

Dysregulation of NM in cancer cells commonly coincides with metabolic reprogramming (Fig. 1; [32]). An increase in nucleotide availability is a key contributor to cancer initiation and progression since cancer cells rely heavily on DNA and RNA synthesis for rapid growth and proliferation. Increasing evidence suggests that there is a strong interplay between dysregulated NM, the composition of the IME and the anti‐tumour immune response [34, 56].

## The interplay between nucleotide metabolism and the IME of cancer cells

Purine metabolism is the most well‐studied interaction between NM and cancer immunity. Purinergic signalling, a concept coined by Geoffrey Burnstock in 1970 [72] is induced by purine analogues such as adenosine or extracellular ATP, and results in the induction or inhibition of the immune response. Adenosine is frequently elevated in solid tumours and exerts immunosuppressive activity. Adenosine receptors regulate cytokine production in macrophages, primarily mediated by the A2A receptor [73]. The adenosine receptors are G‐protein coupled and comprise of A1, A2A, A2B, and A3. Upon macrophage activation, adenosine receptors modulate cytokine production by inhibiting tumour necrosis factor alpha (TNF‐α) production, which is mediated primarily by A2A receptor [74, 75]. Furthermore, activation of adenosine receptors in neutrophils regulates ROS production and phagocytosis [74, 76]. The hypoxic tumour environment drives the expression of the transcription factor HIF1α and hence drives the expression of CD39 and CD73. CD39 catalyses the conversion of ADP and ATP into AMP, while CD73 irreversibly catalyses the conversion of AMP into adenosine in different tumour cells including stromal cells, MDSC, and Treg cells. The accumulation of adenosine in the TME is mostly the outcome of metabolising extracellular ATP to adenosine by CD39, CD73 and CD38 [73]. In injuries or tumours in which inflammation, necrosis and apoptosis take place, increased levels of ATP function as a pro‐inflammatory signal. Moreover, extracellular ATP can act on P2 receptors leading to enhanced antigen presentation and a pro‐inflammatory response. In contrast, ATP can also elicit immune suppressive activity by inducing T‐helper 2 and Treg cell differentiation [73].

The interaction between NM in the pyrimidine pathway and cancer immunity is less well characterised, however a recent study demonstrated that pyrimidine NM regulates the mtDNA‐dependent innate immunity, indicating a novel interaction pathway between NM and the immune system [77]. This interaction is driven by YME1L, a mitochondrial protease that regulates and preserves pyrimidine pools by supporting glutamine synthesis and the proteolysis of the pyrimidine transporter SLC25A33. YME1L deficiency in *YME1L*−/− mice was shown to drive mtDNA inflammatory response via cGAS–STING–TBK1 pathway, whereas adding exogenic pyrimidine suppresses it. In addition, inhibiting *de novo* pyrimidine synthesis and hence pyrimidine pool depletion in wild‐type cells induces mtDNA‐driven immune response [77]. Moreover, it has been demonstrated that urea cycle dysregulation (UCD), a metabolic hallmark of numerous cancers, increases pyrimidine production and promotes CAD activation via changes in nitrogen metabolism [78]. Increasing pyrimidine levels also drives purine to pyrimidine transversion and pyrimidine‐rich transversion mutational bias (PTMB) leading to an increase in immunogenicity [78, 79]. A study found that when compared to normal plasma, melanoma tumour interstitial fluid had higher concentrations of the mono and di‐phosphates guanosine di‐phosphate (GDP) and uridine di‐phosphate (UDP) and that this was associated with an increase in CD4^+^, CD25^+^, FOXP3‐, nuclear factor‐kappa B (NF‐_k_B), and an increase in the cytokines; interferons, T‐bet, and IL17, and a decrease in IL13 expression. The authors additionally found that UDP‐treated B16 melanoma cells‐engrafted C57BL6 mice showed a high expression of anti‐tumour immune response markers; tumour‐infiltrating T‐lymphocytes (TILs) CD3^+^ CD8^+^, and CD3^+^CD4^+^, and the major histocompatibility class II high tumour‐associated macrophages (MHC II)^HI^ TAM, which are associated with tumour suppression in early tumour development [80].

## Altered nucleotide metabolism directly regulates immune cell function in the TME

In the complex milieu of the TME, nucleotide metabolism exerts a profound influence on various immune cells, modulating their function and shaping the cancer immunity landscape. The intersection of nucleotide metabolism with immune cell functionality is a critical aspect of cancer progression and therapeutic response.

### T Lymphocytes

T lymphocytes, particularly cytotoxic T‐cells, are pivotal in anti‐tumour immunity [81]. The proliferation and function of these cells are highly dependent on adequate nucleotide supplies for DNA synthesis during clonal expansion [82]. In the TME, cancer cells often monopolise nucleotide resources, leading to a state of nucleotide deprivation for T‐cells. This scarcity can impede T‐cell receptor signalling and subsequent T‐cell activation and proliferation, thereby dampening the anti‐tumour immune response. Moreover, the upregulation of enzymes like indoleamine 2,3‐dioxygenase (IDO) by tumour cells can deplete tryptophan, an essential amino acid for T‐cell function, further exacerbating this effect [83].

### Myeloid‐derived suppressor cells

Myeloid‐derived suppressor cells are a heterogeneous group of immune cells known for their immunosuppressive activities in cancer. The adenosine NM pathway plays a crucial role in the expansion and function of MDSCs in the TME [84]. Elevated extracellular adenosine levels, often a result of increased ATP degradation by ectonucleotidases like CD39 and CD73 expressed on tumour cells, can enhance the immunosuppressive capacity of MDSCs [85]. Adenosine signalling through A2A receptors on MDSCs leads to the production of immunosuppressive molecules like interleukin‐10 (IL‐10) and TGF‐β, which further inhibit T‐cell activity [86].

### Dendritic cells

Dendritic cells are crucial for antigen presentation and the initiation of immune responses. The availability of nucleotides influences DC maturation [87] and in a nucleotide‐depleted environment, as often seen in the TME, DCs may exhibit impaired antigen presentation capabilities. Additionally, adenosine signalling can skew DCs towards a more tolerogenic phenotype, reducing their ability to activate T‐cells and thereby facilitating tumour escape from immune surveillance [88].

### NK cells

Natural killer are innate immune cells known for their ability to kill cancer cells without prior sensitisation [89]. NM, particularly the balance between ATP and adenosine, can significantly affect NK cell function. High extracellular ATP levels can activate NK cells, enhancing their cytotoxic activity [90]. Conversely, an increase in adenosine concentration, a common feature in many tumours, can suppress NK cell‐mediated cytotoxicity and cytokine production [90].

### Tumour‐associated macrophages

Tumour‐associated macrophages are key components of the TME and can exhibit either pro‐inflammatory (M1) or anti‐inflammatory (M2) phenotypes. NM, particularly purinergic signalling, can influence the polarisation of TAMs [91]. For instance, adenosine can drive TAMs towards an M2‐like, tumour‐promoting state, contributing to immunosuppression and angiogenesis within the TME.

The intricate relationship between NM and immune cell dynamics within the TME is a pivotal aspect of tumour immunology. Understanding these molecular pathways in NM offers potential therapeutic avenues to modulate and enhance the immune response against cancer [79, 92].

## Therapeutic modulators of pyrimidine metabolism

Since cancerous cells depend on nucleotide availability for rapid and dysregulated proliferation, targeting NM represents a major vulnerability and therapeutic strategy. Inhibitors and modulators of purine and pyrimidine synthesis pathways have been in clinical use since the 1940s, and remarkably remain the standard‐of‐care treatment in many cancer types today [93, 94].

There are two main treatment modalities which target NM. The first is the use of nucleotide analogues, and the second involves inhibitors or modulators of metabolic enzymes involved in NM. Since many clinical approaches are currently based on targeting pyrimidine metabolism in standard‐of‐care treatments, and emerging clinical efforts build upon this foundation in immunotherapy combinational studies, pyrimidine‐based clinical strategies will be the focus for the remainder of this review.

**Fig. 3:**
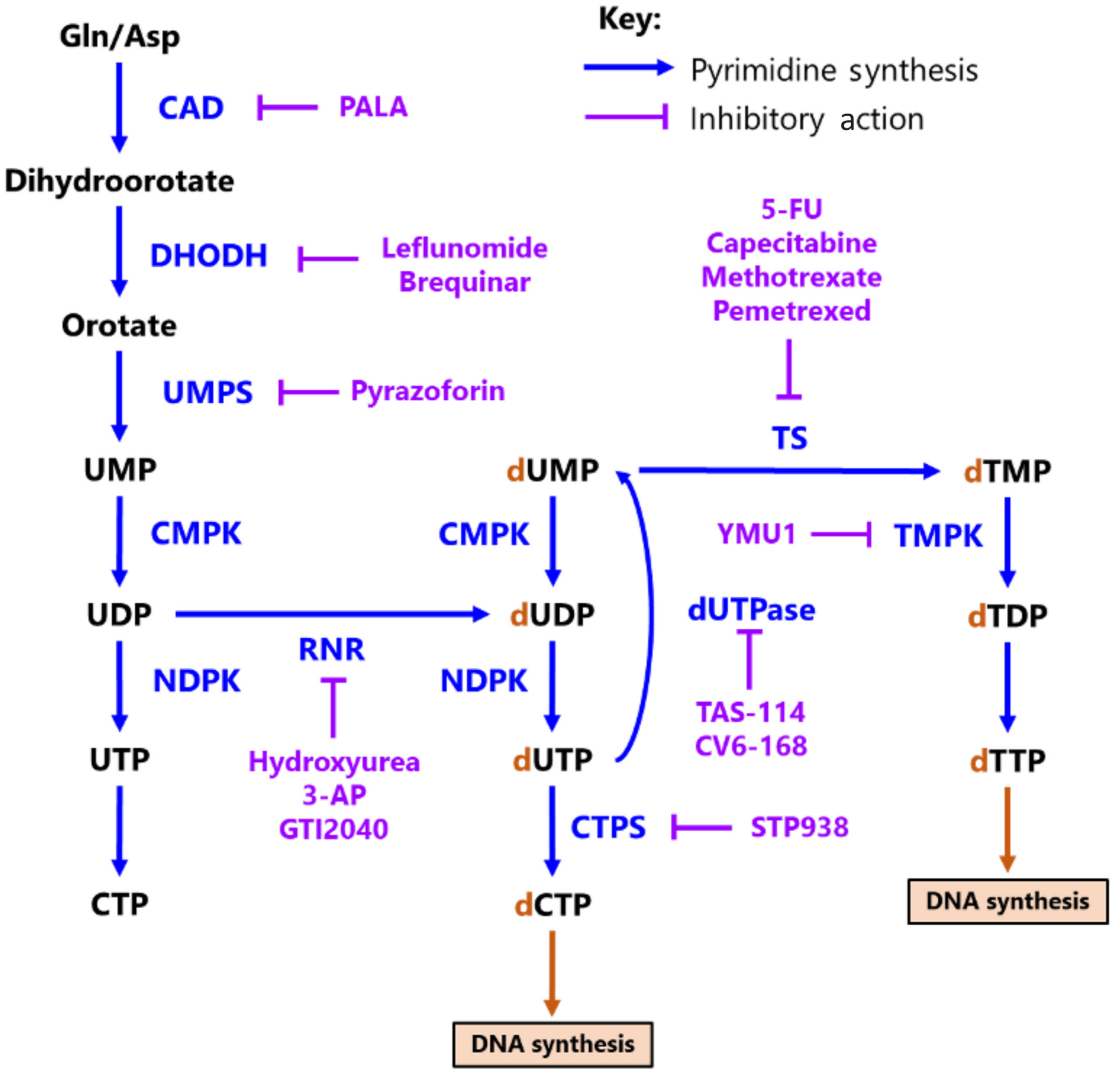
Inhibitors of *de novo* pyrimidine synthesis. Summary pathway depicting enzymatic inhibitors (purple) of key metabolic reactions within *de novo* pyrimidine synthesis. 5‐FU, 5‐fluorouracil; Asp, aspartate; CAD, carbamoyl‐phosphate synthetase 2, aspartate transcarbamylase and dihydroorotase; CMPK, cytidine monophosphate kinase; CTP, cytidine triphosphate; CTPS, CTP synthase; d, ‘deoxy’; DHODH, dihydroorotate dehydrogenase; dUTPase, deoxyuridine triphosphate nucleotidohydrolase; Gln, glutamine; NDPK, nucleotide diphosphate kinase; PALA, *N*‐phosphonoacetyl‐l‐aspartate; TDP, thymidine diphosphate; TMP, thymidine monophosphate; TMPK, thymidine monophosphate kinase; TS, thymidylate synthase; TTP, thymidine triphosphate; UDP, uridine diphosphate; UMP, uridine monophosphate; UMPS, UMP synthetase; UTP, uridine triphosphate.

Modulators of pyrimidine metabolism include pyrimidine analogues and enzymatic modulators of the pyrimidine synthesis pathway (Fig. 3). Synthetic pyrimidine analogues can mimic the activity of the naturally occurring compounds. They interfere with conventional NM by interacting with key metabolic enzymes including kinases, ribonucleotide reductase (RNR), nucleoside phosphorylases and thymidylate synthase. This leads to their assimilation or misincorporation into DNA and RNA, inhibiting canonical replication and repair of genetic material, stunting cell growth and proliferation. Enzymatic modulators of pyrimidine metabolism include direct inhibition of rate‐limiting reactions within pyrimidine biosynthesis, as well as disruptors of other critically linked pathways such as one‐carbon (1C) metabolism, encompassing both folate and methionine metabolism [95]. The two major classes of pyrimidine modulators (fluoropyrimidines and antifolates) fall into both categories, as many nucleotide analogues can have additional metabolic effects which contribute to their overall cytotoxicity [96, 97]. Therapeutic agents targeting *de novo* pyrimidine synthesis are summarised in Fig. 3.

## Fluoropyrimidines

Among the earliest pyrimidine analogues approved for clinical use is 5‐fluorouracil (5‐FU). 5‐FU was synthesised by Heidelberger *et al*. in 1957 and approved for clinical use in 1960 [98] (Table 2). Since then, 5‐FU has remained one of the most successful chemotherapeutic agents, forming the basis for standard‐of‐care therapy in multiple cancer indications including colorectal [105], breast [106], head and neck [107], gastric [108] and pancreatic [109]. Oral 5‐FU pro‐drugs were later synthesised and are now in clinical use. One such example is capecitabine, the first oral chemotherapy approved for the treatment of metastatic colorectal cancer, also now used in breast and head and neck cancers [110]. As fluorinated pyrimidines, 5‐FU and capecitabine exert anti‐cancer activity through inhibition of thymidylate synthase (TS) and exploiting DNA polymerases' inability to distinguish between genotoxic fluorouracil and normal thymine when synthesising DNA [111]. TS is the enzyme responsible for the rate‐limiting step in *de novo* synthesis of thymine, the inhibition of which leads to nucleotide pool imbalances, thymine depletion and a thymine‐less cell death [112]. This potent mechanism of action combined with their high tolerability among patients make TS‐targeted therapies the most widely used class of chemotherapeutics, foundational to numerous combination treatment regimens [93].

**Table 2:** Approved chemotherapeutic modulators of pyrimidine metabolism. 5‐FU, 5‐fluorouracil; Ara‐C, cytarabine; DNA, deoxyribonucleic acid; FUdR, floxuridine; RNA, ribonucleic acid; TS, thymidylate synthase.

Pyrimidine analog	Mechanism of action	Cancer type(s)	Year of approval	Reference(s)
5‐Fluorouracil (5‐FU)	TS inhibition, uracil analogue, DNA misincorporation, RNA misincorporation	Colorectal, Breast, Pancreatic, Head and neck	1956	[96, 98]
Cytarabine (Ara‐C)	Deoxycytidine analogue, DNA misincorporation	Acute non‐lymphocytic leukaemia	1969	[99]
Capecitabine	5‐FU pro‐drug, TS inhibition, DNA and RNA misincorporation	Colorectal, Breast	1998	[100, 101]
Floxuridine (FUdR)	TS inhibition, metabolised to fluorouracil	Colorectal, Liver	1970	[102]
Gemcitabine	Deoxycytidine analogue, DNA misincorporation	Non‐small cell lung cancer, Breast, Ovarian, Pancreatic	1996	[103]
Trifluridine/Tipiracil (TAS‐102)	*Trifluridine*: TS inhibition; thymidine analogue; DNA misincorporation *Tipiracil*: thymidine phosphorylase inhibitor	Colorectal	2012	[104]

## Antifolates

Antifolates block the synthesis of folate co‐factors needed for normal pyrimidine and purine biosynthesis. Methotrexate was among the first ever curative chemotherapeutics to be discovered, inducing complete remission in acute lymphoblastic leukaemia (ALL) in the late 1940s [113]. Currently, it is administered to ALL patients with central nervous system metastases but has also been used to treat solid tumours [114]. Pemetrexed, another antifolate that was approved relatively recently in 2004. It is used alongside carboplatin or cisplatin as a standard‐of‐care treatment for non‐small cell lung cancer (NSCLC) and mesothelioma respectively [115, 116]. Antifolates are unique to other modulators of pyrimidine metabolism as they have a dual function in targeting both pyrimidine and purine synthesis. In addition to TS, methotrexate and pemetrexed inhibit dihydrofolate reductase (DHFR), with pemetrexed also inhibiting glycinamide ribonucleotide transformylase (GART), a key mediator of purine metabolism. Although the predominant cytotoxic mechanism of pemetrexed is through TS inhibition [117], an early study demonstrated that *in vitro* cytotoxicity of pemetrexed could not be completely rescued solely by restoration of pyrimidine nucleotide pools, but required additional restoration of purine pools [118]. This suggests that this dual mechanism of pyrimidine and purine modulation is critical for its anti‐cancer activity. Table 2 shows a list of commonly used approved pyrimidine analogues that target different aspects of the pyrimidine metabolism pathway.

## Inhibition of pyrimidine metabolism changes the IME landscape

Targeted inhibition of pyrimidine metabolism can stimulate immune cell infiltration within the TME. Several studies have shown that 5‐FU can influence the TME, particularly the immunological microenvironment. Studies using colon and melanoma tumour models demonstrated the role of 5‐FU in lowering tumour burden by activating the cancer cell's intrinsic cyclic GMP‐AMP synthase and the cyclic GMP‐AMP receptor stimulator of interferon genes (cGAS‐STING) pathway, which in turn triggers anti‐tumour activity. The cGAS‐STING pathway senses the cytosolic DNA and induces IFN I expression [119]. In addition, 5‐FU induced the production of IFN I, and T‐cell infiltration, and was found that T‐cell depletion reduced the efficacy of 5‐FU [120]. Low doses of 5‐FU were also shown to cause significant MDSC depletion in the blood and the spleen of the Lewis lung carcinoma (LLC) mouse model, resulting in a reduction of tumour volume due to the increased levels of cytotoxic T‐cells [121].

Pemetrexed has also been reported to play an important role in TME immune regulation. Treatment with pemetrexed induced the expression of programmed cell death ligand 1 (PD‐L1) in NSCLC [122]. PD‐L1 is an immune checkpoint protein that regulates T‐cell activity, particularly in suppressing autoimmune responses. It binds PD‐1 on T‐cells, resulting in T‐cell inhibition and exhaustion [122]. Moreover, in a mouse model of colorectal cancer, pemetrexed activated ICD, which in turn promoted the activation of several immune associated genes [123]. A dual role for pemetrexed that involves activation of CD4^+^ infiltrating cells and APCs, as well as promoting PD‐L1 expression facilitating the activation of cytotoxic T‐cells by the action of ICB therapy [124]. The same study highlighted that TS inhibition increased PD‐L1 expression via the accumulation of ROS which activates the NF‐_k_B‐PD‐L1 pathway [124]. Furthermore, it was found that sequential administration of pemetrexed and cisplatin activated the STING pathway and ICD, and enhanced immune function through elevated levels of CD8^+^ infiltrating cells, as well as PD‐L1 upregulation in comparison to combination therapy in a NSCLC mouse model [125].

## Immunotherapy combination strategies to enhance the efficacy of pyrimidine metabolism modulators

The discovery of the immunomodulatory effects of pyrimidine NM inhibitors has provided strong rationale for the development of novel clinical strategies combining pyrimidine pathway inhibitors with immunotherapy. Immunotherapy was first harnessed by William Coley in the late 19th century, who treated bone sarcomas with bacterial‐based vaccines to induce an anti‐tumour immune response [126]. Their use in modern clinical efforts has expanded significantly in the 21st century due to the discovery and characterisation of immune checkpoints, which led to the development of ICB therapy [127]. The first chemo‐immunotherapy combination strategy combined methotrexate with immunisation and observed an increase in progression‐free survival compared to either single‐agent treatment [128]. Not only did this establish a precedent for chemo‐immunotherapy combination strategies for cancer treatment, but also for combining modulators of NM with immunotherapy. Table 3 contains a summary of disruptors of pyrimidine metabolism in current clinical trials with immunotherapy.

**Table 3:** Modulators of pyrimidine metabolism in current clinical trials with immunotherapy. 5‐FU, 5‐fluorouracil; CAD, carbamoyl‐phosphate synthetase 2, aspartate transcarbamoylase, and dihydroorotase; CRC, colorectal cancer; DHFR, dihydrofolate reductase; DNA, deoxyribonucleic acid; ESCC, oesophageal squamous‐cell carcinoma; GART, glycinamide ribonucleotide transformylase; GEC, gastroesophageal cancer; IDO, indoleamine 2, 3‐dioxygenase 1; NCT, The National Clinical Trial number; NSCLC, non‐small cell lung cancer; PD‐1, programmed death protein‐1; PD‐L1, programmed death ligand‐1; TS, thymidylate synthase.

Drug name	Mode of action	Immunotherapy combinational strategy	Cancer type(s)	Clinical trial phase^a^	NCT identifier
5‐Fluorouracil (5‐FU)	TS inhibition; uracil analogue; DNA misincorporation	Atezolizumab (anti‐PD‐L1)	Rectal cancer	Phase Ib/II, recruiting	NCT03127007
		Nivolumab (anti‐PD‐1)	ESCC	Phase II, recruiting	NCT05213312
		Entrumadenant (anti‐adenosine receptor)	CRC, GEC	Phase I/Ib, completed	NCT03720678
		Tislelizumab (anti‐PD‐1)	Head and neck cancer	Phase II, recruiting	NCT05758389
Pemetrexed	TS inhibition; DHFR inhibition; GART inhibition	Durvalumab (anti‐PD‐L1)	NSCLC	Phase II, recruiting	NCT04163432
		Pembrolizumab (anti‐PD‐1) IDO102 (anti‐IDO, anti‐PD‐L1)	NSCLC	Phase I/II, completed	NCT03562871
		Atezolizumab (anti‐PD‐L1) Dendritic cell vaccination	Mesothelioma	Phase I/II, recruiting	NCT05765084
Gemcitabine	Deoxycytidine analogue; DNA misincorporation	Durvalumab (anti‐PD‐L1) Oleclumab (anti‐CD73)	Pancreatic cancer	Phase Ib/II, completed	NCT03611556
		Dendritic cell vaccination	Pancreatic cancer	Phase I/II, completed	NCT00547144
5‐FU/Pemetrexed		Pembrolizumab (anti‐PD‐1)	Head and neck cancer/advanced lung cancer	Phase II, recruiting	NCT05358548
5‐FU/Gemcitabine		Algenpantucel‐L (whole‐cell immuno‐stimulant)	Pancreatic cancer	Phase III, completed	NCT01072981
Afatinib	CAD inhibition	Pembrolizumab (anti‐PD‐1)	Head and neck cancer	Phase II, completed	NCT03695510

^a^ As of January 2024.

At the molecular level, most clinical investigations have been aimed at improving responses within immune hot tumours, particularly those with high microsatellite instability (MSI) status, given they respond better to immunotherapy [129, 130, 131]. The majority of patients exhibit microsatellite stable (MSS) status and for the most part do not respond to ICB therapies. More recent efforts, however, are focused on identifying MSI and MSS sub‐groups which have a greater likelihood of response to immunotherapy [132]. Combining modulators of NM with immunotherapy in this setting represents a promising clinical strategy to eliminate cancer cells whilst triggering the release of neoantigens to elicit an immune response [133]. This approach could offer significant clinical benefits for a large cohort of patients who would otherwise be ineligible for receiving immunotherapy.

## Conclusion and future perspectives

The investigation of the TME continues to be a fertile ground for novel insights, particularly in the context of cancer metabolism and immune interactions. The reprogramming of cancer metabolism, including NM, is a cornerstone in supporting the aggressive growth and proliferation characteristics of cancer. This metabolic reshaping extends beyond the cancer cells themselves, exerting profound influences on the IME. The capacity of cancer cells to modulate and even undermine the host's immune defences plays a critical role in sustaining tumour growth and facilitating metastasis.

Recent studies have shed light on the intricate relationship between pyrimidine metabolism and the IME. Emerging evidence suggests that strategic modulation of pyrimidine metabolism can enhance T‐cell infiltration into the TME, bolstering the inflammatory response against the tumour. This modulation appears to counteract the tumour's immune evasion tactics, thereby reinstating the host immune system's ability to mount an effective anti‐tumour response. This insight not only provides a clearer understanding of the dynamic interactions within the TME, but also opens new vulnerabilities for therapeutic interventions.

In the field of therapeutic strategies, there is a growing trend towards leveraging the immunomodulatory effects of NM modulation. Specifically, combining pyrimidine metabolism modulators with established immunotherapies holds significant promise. This approach aims to synergise the direct anti‐tumour effects of NM modulators with the enhanced immune response elicited by immunotherapy. Such combinations could potentially improve the efficacy of standard‐of‐care treatments across various cancer types, offering a more personalised and effective approach to cancer therapy.

## Conflict of interest

RDL, KAM and PMW are CV6 Therapeutics employees. MJL is a CV6 Therapeutics Consultant. HS and AE declare that there are no competing interests.

## Author contributions

The conception and design of the work were initiated by MJL. The drafting of the manuscript was collaboratively undertaken by HS and AE, who both contributed significantly to the development and articulation of the primary content. Subsequent critical revisions, aimed at enhancing the intellectual content and ensuring accuracy, were performed by PMW, RDL, KAM and MJL. All mentioned authors (HS, AE, PMW, KAM, RDL and MJL) have also reviewed and approved the manuscript in its final form.
